# Reversing Cognitive Decline in Aging: Reversible Mechanistic Defects and a Novel Nutritional Intervention

**DOI:** 10.1093/geroni/igaa057.3156

**Published:** 2020-12-16

**Authors:** Rajagopal Sekhar, George Taffet

## Abstract

Aging is the biggest risk factor for cognitive-decline and Alzheimer’s disease (AD), but underlying mechanisms are not well-understood and interventions are lacking. Cognitive-decline in AD has been associated with deficiency of glutathione, (the most abundant, intracellular, antioxidant protein), elevated oxidative-stress, insulin-resistance and increased inflammation. We identified and reported that glutathione-deficiency and oxidative-stress in older-adults occur due to decreased availability of precursor amino-acids glycine and cysteine, and can be corrected with GlyNAC (a combination of glycine and the cysteine precursor N-acetylcysteine). We hypothesized that cognitive decline in older-adults is linked to glutathione-deficiency, mitochondrial-dysfunction, oxidative-stress, insulin-resistance, and inflammation. The first abstract discusses the rationale and findings of an open-label clinical trial: compared to young-humans, older-adults had cognitive-decline, glutathione-deficiency, mitochondrial-dysfunction, abnormal glucose-metabolism and insulin-resistance, oxidative-stress, endothelial-dysfunction and inflammation. These defects were improved/reversed by supplementing GlyNAC for 24-weeks, but benefits receded on stopping GlyNAC for 12-weeks. The second abstract presents a study in 8 young (20-weeks old) and 16 aged (90-weeks old) wild-type male C57BL/6J mice where we found that aged-mice had naturally-occurring cognitive-impairment, and brain defects in glutathione-deficiency, oxidative-stress, glucose-transport, mitochondrial glucose-oxidation, insulin-resistance, endoplasmic-reticulum stress, autophagy, mitophagy, inflammation, senescence, genomic and telomere damage. Aged-mice received either GlyNAC or isonitrogenous-placebo supplementation for 8-weeks, and only GlyNAC-fed mice improved cognition and brain defects. Collectively these data highlights the discovery of novel and reversible mechanistic defects in older-adults and aged-mice with naturally-occurring cognitive-decline, and identifies that supplementing GlyNAC can improve brain-health and cognition. These findings could have important implications for reversing cognitive-decline in older-adults, and AD.

